# Potential Antimicrobial and Anticancer Activities of an Ethanol Extract from *Bouea macrophylla*

**DOI:** 10.3390/molecules25081996

**Published:** 2020-04-24

**Authors:** Ngoc Hong Nguyen, Thuy Trang Nguyen, Phu Cuong Ma, Qui Thanh Hoai Ta, Thuc-Huy Duong, Van Giau Vo

**Affiliations:** 1 CirTech Institute, Ho Chi Minh City University of Technology, Ho Chi Minh City 700000, Vietnam; nn.hong@hutech.edu.vn; 2 Faculty of Pharmacy, Ho Chi Minh City University of Technology, Ho Chi Minh City 700000, Vietnam; nt.trang85@hutech.edu.vn; 3 Institute of Applied Science, HCMC University of Technology (HUTECH), Ho Chi Minh City 700000, Vietnam; maphucuong1999@gmail.com; 4 Institute of Research and Development, Duy Tan University, Danang 550000, Vietnam; tathoaiqui@duytan.edu.vn; 5 Department of Organic Chemistry, University of Education, Ho Chi Minh City 700000, Vietnam; huydt@hcmue.edu.vn; 6 Bionanotechnology Research Group, Ton Duc Thang University, Ho Chi Minh City 700000, Vietnam; 7 Faculty of Pharmacy, Ton Duc Thang University, Ho Chi Minh City 700000, Vietnam

**Keywords:** *Bouea macrophylla*, extract, antimicrobial activity, HeLa, HCT116

## Abstract

*Bouea macrophylla* is a tree widely grown throughout South East Asia. It is used in folk medicine for the treatment of various illnesses. The present study aimed to identify the chemical constituents and to test the antimicrobial and anticancer activities of an ethanol extract from *B. macrophylla* leaves. The extract exhibited excellent antibacterial properties against 9 out of 10 target microorganisms. including four Gram-negative bacteria (*Escherichia coli, Shigella flexneri, Vibrio cholera,* and *Pseudomonas aeruginosa)* and four Gram-positive bacteria (*Staphylococcus aureus, Listeria monocytogenes, Enterococcus faecalis,* and *Bacillus cereus*), as well as a fungus (*Candida albicans).* In addition, the extract was also tested on HeLa and human colorectal carcinoma (HCT116) cells to evaluate its cytostatic effects. The ethanol extract was able to inhibit the proliferation of HeLa and HCT116 cells, showing IC_50_ = 24 ± 0.8 and 28 ± 0.9 µg/mL, respectively, whereas the IC_50_ values of doxorubicin (standard) were 13.6 ± 1.3 and 15.8 ± 1.1 µg/mL respectively. Also, we identified various bioactive compounds in the extract such as polyphenols, flavonoids, caryophyllene, phytol, and trans-geranylgeraniol by GC-MS, which could contribute to the extract’s biological activities. Therefore, our findings strongly indicate that the constituents of the *B. macrophylla* ethanol extract could be active against the tested bacteria and fungi as well as cancer cells. Further investigation is needed to understand the mechanisms mediating the antimicrobial and anticancer effects and identify signaling pathways that could be targeted for therapeutic application.

## 1. Introduction

Plants are considered a valuable source of unique natural compounds for the development of antidiabetic, anti-inflammatory, anticancer, and antimicrobial drugs [1]. There is evidence that minor components of food such as phytochemicals play a significant role in the reduction of the incidence of many chronic diseases [2,3]. The consumption of foods rich in phytochemicals and other bioactive food components has been clearly linked to the prevention and reduction of cancer and cardiovascular diseases and to the improvement of the immune system function [4]. Although a large number of natural products have been discovered and studied recently, the search for new natural compounds with antimicrobial and antioxidant activities still needs further effort.

*Bouea macrophylla* (or *Bouea gandaria* Blume, *Bouea burmanica* Griff.), commonly known as marian plum, plum mango, gandaria, or maprang, is a tropical fruit that is widely grown throughout South East Asia, particularly in the countries of Thailand, the Philippines, Malaysia, and Indonesia. *B. macrophylla* (belongs to the same family as mango (Anacardiaceae), but its taste is notably different [5]. It is regarded as a fruit that can benefit health by providing a range of vitamins, minerals, and fibers, which may exert a wide range of pharmacological activities linked to their antioxidant, anticancer, antimicrobial, and anti-inflammatory properties [5,6].

In Vietnam, various parts of *B. macrophylla* such as leaves, flowers, fruits, seeds are used as food and may be beneficial to health due to their phytochemical bioactive compounds. The antioxidant activity of ethanol extracts from the leaves of *B. macrophylla* has been reported [7] and may help to prevent several diseases such as cancer and Alzheimer’s disease [8,9,10,11]. However, there is still limited information on the chemical constituents of *B. macrophylla* which exert biological activity. The aim of the present study was to determine both the antimicrobial activity against different pathogenic bacteria and the antiproliferative effects on human cancer cell lines of a *B. macrophylla* ethanol extract, after identifying its bioactive compounds by gas chromatography–mass spectrometry (GC-MS).

## 2. Material and Methods

### 2.1. Chemicals and Reagents

The compounds 2,4,6-Tris(pyridyl)-s-triazin (TPTZ), 2,2-diphenyl-1-picrylhydrazyl (DPPH), ciprofloxacin, ethanol, gallic acid, and rutin were purchased from Sigma-Aldrich (St. Louis, USA). Dimethyl sulfoxide (DMSO), Folin–Ciocalteu reagent, and methanol were obtained from Merck (Darmstadt, Germany). Aluminum chloride and iron (III) chloride were purchased from BDH (Cambridge, England). Luria Bertani broth (LB broth) and Mueller–Hinton Agar (MHA) were purchased from BD-Difco (BD Biociences, USA), Ascorbic acid was from MP Biomedicals (Illkirch, France). Doxorubicin hydrochloride (Sigma, St. Louis, MO) was the standard drug used as positive control in this study. All the other chemicals were of analytical grade.

### 2.2. Extraction of a Crude Extract of B. macrophylla

Fresh leaves of *B. macrophylla* were collected at O Mon district, Can Tho city, in the South of Vietnam (October–November 2018). Botanical identification was confirmed by Assoc. Prof. Tran Hop. Ho Chi Minh City University of Natural Science, Vietnam. The leaves were triple-washed with tap water, dried with hot air at 60 °C, and then crushed to grit (1.0 mm size) for further extraction. Subsequently, the dried material (200 g) was macerated in 70% ethanol (3 × 2 L) for 24 h at room temperature with shaking at 150 rpm. The filtered solution was evaporated to dryness under reduced pressure at 40 °C at an approximate rotation speed of 200 rpm using a rotary evaporator (Heidolph, Germany) to obtain a crude extract (26.8 g). Then, this crude extract was further used to analyze the bioactivity. Prior to the bioactivity assay, the crude extract was dissolved in DMSO (10% final concentration) and filter-sterilized with a 0.2 µm membrane filter (Millex) to make a stock solution.

Phytochemical groups were identified in the sample using standard procedures [12] improved by the Department of Pharmacognosis, Faculty of Pharmacy, University of Medicine and Pharmacy, Ho Chi Minh City [13].

### 2.3. Determination of Total Phenolic Content

The total phenolic content of the ethanol extract was assessed using the Folin-Ciocalteu method with slight modifications [14]. The amount of total phenols was determined based on mg of gallic acid equivalent per gram of sample (mg GAE/g).

### 2.4. Determination of Total Flavonoid Content

The total flavonoid content was estimated using a previously reported method by Formagio (2014) [15]. The total content of flavonoids was measured as mg of rutin equivalents per gram of sample (mg RE/g), using a rutin calibration curve.

### 2.5. Free-Radical Scavenging Activity Assay

The DPPH assay (2, 2-diphenyl-1-picrylhydrazyl) was performed and evaluated using the procedure proposed by Von Gadow et al. (1997) [16]. We measured the inhibitory percentage of DPPH radicals in the samples according to the formula suggested by Yen and Duh (1994) [17].

### 2.6. Ferric Reducing Antioxidant Power Assay (FRAP)

The total antioxidant potential of the samples was determined using the ferric reducing ability of plasma as a measure of “antioxidant power” (FRAP assay) [18].

### 2.7. GC-MS Analysis

GC-MS analysis of the ethanol extract was carried out using a Hewlett Packard GC system with a GC 5890 Series II/MS-5971A model (Hewlette Packard, Paulo Alto, CA, USA,) equipped with a DB-5MS column (30 m in length × 250 μm in diameter × 0.25 μm in thickness of film). Spectroscopic detection by GC–MS was associated with an electron ionization system (70 eV). Pure helium gas (99.995%) was used as the carrier gas. with a column head pressure of 50 kPa. The oven was initially heated to 70 °C, held at this temperature for 5 min, then the temperature was ramped up to 300 °C at 10 °C/min and held at this final value for 10 min prior to finishing. The relative quantity of the chemical compounds identified in the ethanol extract of *B. macrophylla* was expressed as a percentage dependent on the chromatogram peak area. The chemical constituents of the ethanol extract were detected based on their GC retention time in the DB-5MS column, using standard computer software data matching of the spectra (NIST MS Search 2.0).

### 2.8. Agar Well Diffusion Method for the Determination of the Antimicrobial Activity of the Leaf Extract

A total of 10 pathogenic bacterial strains were supplied by Ho Chi Minh City University of Science, including *Escherichia coli* ATCC 11775, *Shigella flexneri* ATCC 12022, *Vibrio cholera* ATCC 17802, *Pseudomonas aeruginosa* ATCC 9027, *Staphylococcus aureus* ATCC 6538, *Listeria monocytogenes* ATCC 15313, *Bacillus cereus* ATCC 11778, *Enterococcus faecalis* ATCC 29212, *Streptococcus mutans* ATCC 25175, and *Candida albicans* ATCC 90028. The bacteria were grown overnight at 37 °C in Luria Bertani broth (Merck). The strains were grown in liquid medium to an optical density (OD_600nm_) of ~0.8–1.0 (corresponding to approximately 1 × 10^8^ colony-forming unit (CFU)/mL. ascertained by plate counts on nutrition agar). Antibacterial screening was performed by the agar well diffusion method, as previously described [19]. Briefly, a 0.1 mL suspension of each microorganism containing around 1 × 10^8^ CFU/mL was spread evenly over Mueller–Hinton Agar (BD-Difco). Wells of 6 mm in diameter were punched in the culture media with a sterile cork borer, as previously described [20]. Then, 100 µL of the aliquots ranging from 0.01 to 500 mg/mL was dropped into each well to fullness. The treated dish was incubated at 37 °C for 24 h. The antimicrobial activity was evaluated by determining the diameter of the inhibition zone. Ciprofloxacin (500 µg/mL) and 5% DMSO in distilled water were used as positive and negative reaction controls, respectively.

### 2.9. Cell Culture and Cytotoxicity Assay

HeLa and human HCT116 colorectal carcinoma (HCT116) cell lines were cultured in high-glucose Dulbecco’s Modified Eagle Medium (DMEM) (Sigma) supplemented with 10% fetal bovine serum (FBS) (Gibco) and 1% penicillin/streptomycin and incubated at 37 °C in a humidified incubator in an atmosphere of CO_2_ (5%). The cells were seeded in a 96-well plate at a density of 1 × 10^5^ cells/well and incubated for 24 h at 37 °C in a CO_2_ incubator. The day after, the medium was replaced with fresh medium containing the extract solution at different concentrations corresponding to 100, 50, 25, 12.5, 6.25, 3.15, and 1.6 µg/mL. After incubation for 48 h, the medium was removed, followed by washing with PBS for three times, and 100 µL of fresh medium was added into each well. After another 30 min of incubation to equilibrate to room temperature, 100 µL of CellTiter-Glo^®^ Luminescent reagent (Promega) was added to each well to measure cell viability using a microplate reader (Perkin Elmer, Victor X5, USA). Doxorubicin hydrochloride was used as a positive control, and 1% DMSO in growth media was used as a negative control. The percentage of cell viability was calculated based on changes in luminescence absorbance by comparing the absorbance values between the treated samples and the control wells. The experiments were repeated three times for statistical analysis. To further evaluate the morphological changes of the cells, an optical microscopy was used.

### 2.10. Statistical Analysis

The experiments were performed in triplicate. The data are expressed as mean ± standard deviation. The data were analyzed using the Statistical Package for Social Sciences (SPSS) (version 20.0) software. A *p* value of less than 0.05 was considered significant.

## 3. Results

### 3.1. Phytochemical Screening

The phytochemical study of the ethanol leaf extract revealed the presence of various bioactive compounds (Table 1) including polyphenols, tannins, flavonoids, carbohydrates, steroids, triterpenoids, fixed oils, saponins, and alkaloids, similar to what previously reported for Malaysian *B. macrophylla* [21]. The phytochemical analysis revealed a high total phenolic content of the *B. macrophylla* extract, corresponding to 88.78 ± 6.51 mg GAE/g and a flavonoid content of 70.66 ± 7.51 mg RE/g. A quantitative phytochemical analysis showed that the ethanol extract contained a significant amount of phenolic and flavonoid compounds, confirming its antioxidant properties. Therefore, according to the results, *B. macrophylla* contains active compounds with potential anticancer and antimicrobial activities.

### 3.2. Metabolite Profiling

**Figure 1:**
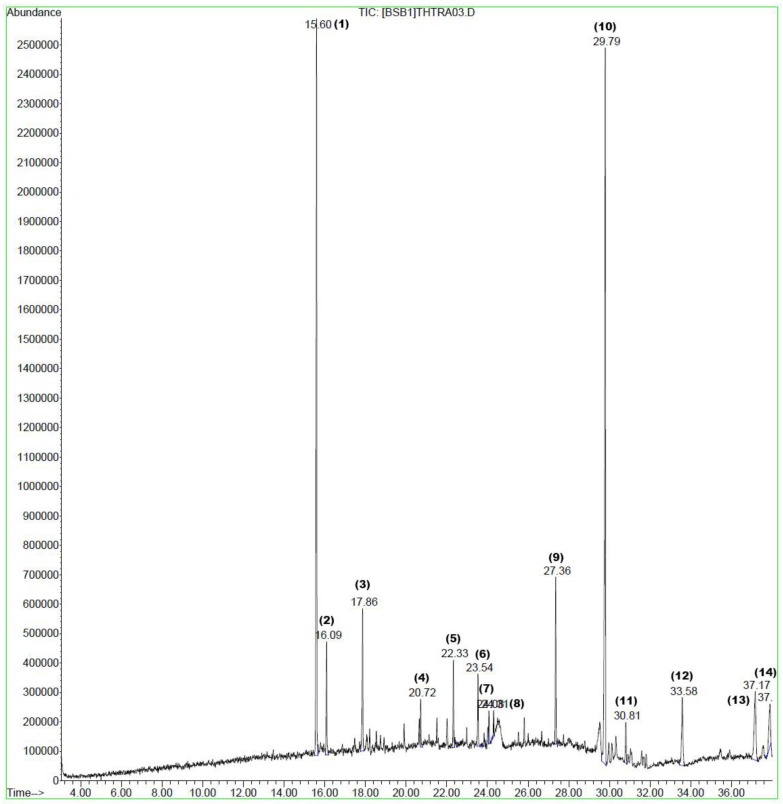
Chromatogram of the *B. macrophylla* extract. The x-axis indicates the retention time in minutes, while the y-axis indicates the peak % signal intensity.

In total, 14 compounds were putatively identified in the *B. macrophylla* extract using GC–MS analysis (Table 2). An optimized method produced the GC chromatogram shown in Figure 1, where the y-axis represents the % signal intensity, and the x-axis indicates the retention time in minutes (see also Figures S1–S14). Plants continue to be the source of natural pharmacologically active compounds with diverse structures with potential for the treatment or prevention of various diseases. Table 2 lists the compounds from *B. macrophylla* extract identified by GC–MS with putative biological activity, in agreement with previous studies [21,22,23,24,25,26]. The two major compounds identified are caryophyllene (27.48%) and squalene (32.11%). Other main compounds are humulene, caryophyllene oxide, ethyl hexadecanoate, phytol, diisooctyl phthalate, vitamin E, retinol acetate, and γ-himachalene. The compounds with reported activities in the literature may explain the biological activity exhibited by the extract in this study.

### 3.3. Antioxidant Capacity of B. macrophylla Extract

**Figure 2:**
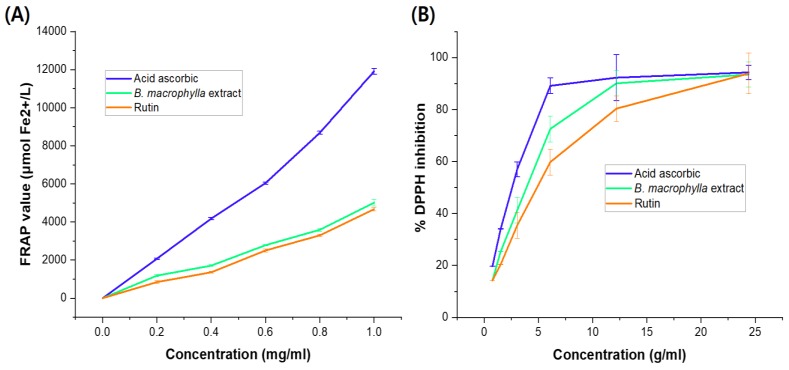
Antioxidant potential determined by Ferric Reducing Antioxidant Power (FRAP) assay (**A**) and DPPH radical-scavenging capacity (**B**) of the *B. macrophylla* extract. Data are shown as mean ± SD of three independent experiments (*p < 0.01; the mean difference is significant at the 0.01 level compared to the control by one-way ANOVA).

The antioxidant capacity of *B. macrophylla* extract determined by the FRAP assay is presented in Figure 2A. As shown by the FRAP value, the plant extract showed strong antioxidant potential, which was 3.01 times lower than that of the standard ascorbic acid and higher than that of rutin (*p* < 0.01) at same concentration of 1 mg/mL. As shown in Figure 2B, the ethanol extract exhibited significant free-radical scavenging activity, as determined by the DPPH assay. The antioxidant effect of this extract was comparable to those of ascorbic acid and rutin, used as reference compounds. The IC_50_ value of the *B. macrophylla* extract (3.94 ± 0.21 µg/mL) was 1.38-fold lower than that of ascorbic acid (2.86 ± 0.11 µg/mL) but 1.24-fold higher than that of rutin (4.90 ± 0.12 µg/mL).

### 3.4. Antibacterial Activity of the Leaf Extract

In the agar diffusion assay, the ethanolic leaf extract of *B. macrophylla* revealed a broad-spectrum antibacterial activity against both Gram positive and Gram-negative bacteria, as well as against a fungus, with zones of inhibition ranging from 11 mm to 24 mm. Among the six concentrations (0.01, 0.01, 1, 10, 100, and 500 mg/mL) of the leaf extract tested, the zone of inhibition shown in 9 out of 10 of the tested microorganism cultures increased with the increase in the concentration of the leaf extract; maximum inhibition was recorded at 500 mg/L of extract (Table 3). The mean inhibition zone for the Gram-negative bacteria ranged from around 11 to 22 mm, indicating a remarkable antibacterial effect when compared with that of ciprofloxacin, used as a positive control, which ranged from 25 to 33 mm. For Gram-positive bacteria, the mean inhibition zone ranged from 11 to 24 mm, while that for the positive control was between 18 and 30 mm.

**Figure 3:**
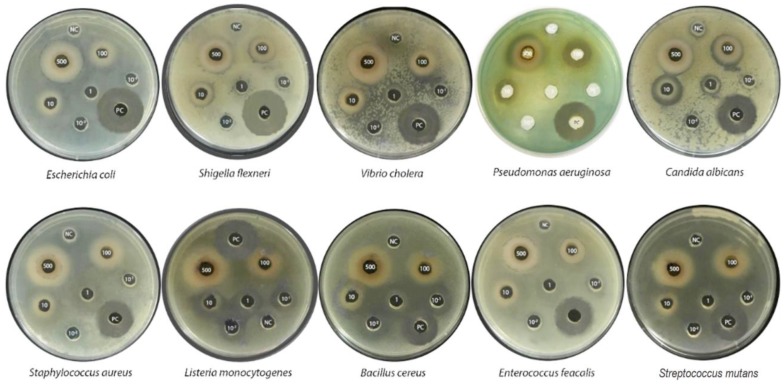
Growth inhibition of some pathogenic bacterial strains caused by the *B. macrophylla* extract at concentrations of 10^−2^, 10^−1^, 1, 10, 100, and 500 mg/mL. PC, positive control (ciprofloxacin); NC, negative control.

The ethanol extract of *B. macrophylla* showed the maximum zone of inhibition against *B. cereus*, ranging from 17 mm to 24 mm, followed by *S. flexneri, V. cholerae, E. coli,* and *C. albicans*; for the other bacteria, the zone of inhibition ranged from 11 mm to 18 mm. Remarkably, *B. cereus* was the most sensitive bacterium to all the concentrations tested (0.01 to 500 mg/mL) of the leaf extract, exhibiting zones of inhibition from 17 mm to 26.0 mm, larger than those of the other bacterial strains tested. Among all the concentrations examined, the maximum zone of inhibition of 24 mm at 500 mg/mL leaf extract of *B. macrophylla* was exhibited by *B. cereus*, and the smallest one (16 mm) was shown by *E. faecalis*, whereas there the extract had no activity against *S. mutans*, even at the highest concentration (Figure 3).

### 3.5. Cytotoxic Activity of the B. Macrophylla Ethanol Extract

**Figure 4:**
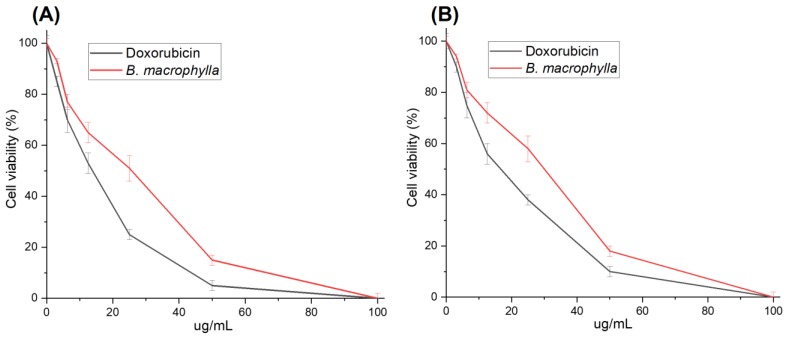
Cell survival curves of HeLa (**A**) and HCT116 (**B**) cell lines treated for 48 h with Doxorubicin and the *B. macrophylla* ethanol extract at difference concentrations. Data are shown as mean ± SD of three independent experiments (* *p* < 0.05; the mean difference is significant at the 0.05 level compared to the control by one-way ANOVA).

**Figure 5:**
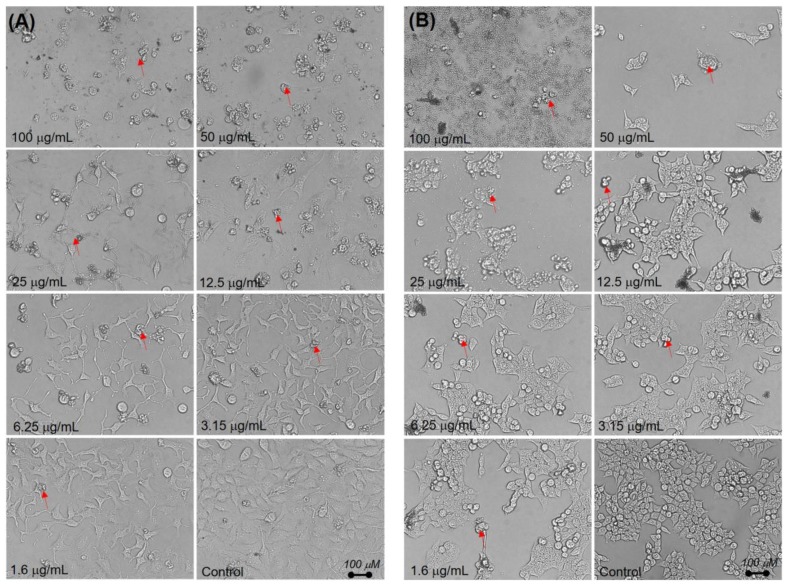
Morphological changes of (**A**) HeLa and (**B**) HCT116 cells treated with the *B. macrophylla* ethanol extract at difference concentrations for 48 h. Morphological changes corresponding to cell shrinkage re indicated by red arrows.

Plant-based medicinal therapeutics are drawing the attention of researchers to develop natural products as potential anticancer drugs [49,50]. To evaluate the potential anticancer activity of the extract, we first compared its in vitro cytotoxicity to that of doxorubicin in HeLa and HCT116 cell lines. Doxorubicin had a relatively higher cytotoxicity potency in HeLa and HCT116 cell lines, with IC_50_ of 13.6 ± 1.3 and 15.8 ± 1.1 µg/mL, respectively (Figure 4A). The *B. macrophylla* ethanol extract showed IC_50_ values of 24 ± 0.8, and 28 ± 0.9 µg/mL in HeLa and HCT116 cells, respectively (Figure 4B). As a standard therapeutic drug, doxorubicin hydrochloride was used in this experiment and showed great toxicity towards the tested cell lines. HeLa cells were more inhibited by the extract compared to HCT116 cells. In addition, the data were supported by morphological observations of the extract-treated cells, showing a significate change in cell morphology characterized by cell shrinkage and cell wall blabbing. Also, there was a clear reduction of the cell populations treated with the higher concentrations of extract compared to untreated cells (Figure 5), typically indicating cytotoxicity and induction of apoptosis in the cells by the extract.

## 4. Discussion

### 4.1. Plant Metabolite Profile of B. Macrophylla Ethanol Extract

Alkaloids, polyphenols, triterpenoids, and saponins that we identified in the B. Macrophylla extract may possess many beneficial bioactivities, such as antioxidant, anti-inflammatory, anticancer, antihyperglycemic, and antidiabetic activities [22]. In addition, alkaloids provide the underlying structure for the development of several antibiotics with a diverse range of action [23]. Phenolic compounds are necessary components of plants, as they can serve as reducing agents, donors of hydrogen, and chelators of metals. Flavonoids possess scavenging or chelating antioxidant activity [24,25]. A previous study also revealed that the leaves of *B. macrophylla* had potent antioxidant activity using the ABTS free-radical scavenging and metal chelating assays [7]. In this study, the results of two assays—FRAP (total electron transfer-based) and DPPH (hydrogen atom transfer-based)—revealed that the plant extract exhibited strong antioxidant activity.

Accurate mass spectrometry was performed, and the obtained spectral data were analyzed according to specific algorithms which provided specific molecular formulas. Table 2 lists the major compounds identified in the ethanol extract of *B. macrophylla* by GC–MS and their biological activities. Several compounds were previously described to have anti-inflammatory activity [28,29], cytotoxicity [28,29], antifungal [27], antioxidant [33,34,35], antimicrobial [37], and neuroprotective activity [32], suggesting the phytopharmaceutical importance of this plant. Remarkably, the compound diisooctyl phthalate (5.232%) was shown to have antimicrobial properties [37,38] and inhibit melanogenesis [39], as firstly reported after its isolation from *Limonium bicolor* [51]. The identification of diisooctyl phthalate in *B. macrophylla* is reported in this study for the first time. The results obtained in this analysis suggest that the ethanol extract of *B. macrophylla* is a valuable repository of bioactive compounds of significant medicinal value.

### 4.2. Antibacterial Activity

The antibacterial activity determined for various concentrations of the *B. macrophylla* extract has not been reported previously [5,7,21]. This study investigates for the first time the antimicrobial properties of a *B. macrophylla* extract in vitro. The ethanolic leaf extract obtained from *B. macrophylla* revealed strong activity against most of the tested bacterial strains. Among the different microorganisms tested, *B. cereus* proved to be the most sensitive to the extract, followed by *L. monocytogenes, V. cholerae, C. albicans, S. flexneri, E. coli, E. faecalis*, and *S. aureus*, while no zone of inhibition was observed for *S. mutans*. Previous studies revealed that various components of plant extracts, such as terpenoids, alkaloids and phenolic compounds, could inhibit the growth of foodborne and spoilage bacteria, disrupting bacteria enzymatic activity and damaging proteins of the microbial cell membrane [52]. The components of *B. macrophylla* here identified which are believed to be the most important for the extract’s biological activity are polyphenols, flavonoids, caryophyllene, phytol, and trans-geranylgeraniol, as reported in Table 2. On the basis of this finding, the ethanol extract of the leaves could be a good candidate in the search of natural antimicrobial agents against infections or diseases caused by the tested microorganisms. Further studies are needed to isolate and characterize the extract’s bioactive compounds for the development of new antibacterial drugs.

### 4.3. Cytotoxic Activity

The assessment of the cytotoxicity of the *B. macrophylla* extract using a cell viability assay] demonstrated activity against cancer cells, with IC_50_ values from 24 to 29 µg/mL. According to the National Cancer Institute, plant extracts with an IC_50_ ≤ 30 µg/mL possess good cytotoxic properties [53] which indicates that the extract demonstrated excellent activity against both cell types. The cytotoxic or antiproliferative activity of the *B. macrophylla* extract may be mediated by its bioactive constituents. This is in line with the observation that natural plants are a source of compounds for the development of novel anticancer medicines [51,52,53]. Our data on the extract cytotoxicity are supported by morphological observations of the extract-treated cancer cells, showing significant changes in cell morphology such as cell shrinkage and cell wall blabbing. Also, the reduction in cell number induced by high concentrations of the extract was clear in comparison to untreated cells (Figure 5), typically indicating cytotoxicity and induction of apoptosis in the cells [54,55].

Concomitant induction of apoptosis and activation of caspase activity lead to changes in both morphological and biochemical characteristics of cells [56,57]. Since a protective role of polyphenols against carcinogenesis is suggested by previous studies based on cell and animal models [25,49,52,54,55], the present results indicate for the first time the potential anticancer activity of a *B. macrophylla* ethanol extract. As presented in Table 2, it has been demonstrated that caryophyllene [29], caryophyllene oxide [30], hexadecanoic acid, ethyl ester [29], phytol [32], squalene [40,41], and trans-geranylgeraniol [42,43] can induce apoptosis and cell cycle arrest in the G1 phase of the cells. Furthermore, since the cytotoxic activity of the plant extract in both cell lines was demonstrated, it is necessary to carry out in the future a bioassay guided study to isolate and characterize the bioactive compounds responsible for this effect and to evaluate their mechanism of action in order to further understand the medicinal effects on this plant against cancer. In our knowledge, this is the first attempt to analyze *B. macrophylla* effects on cell growth in terms of IC_50_. In the future, the evaluation of protein signaling pathways as well as of the specific activity of the active compounds of this natural extract could be carried out to really understand its potential anticancer properties.

## 5. Conclusions

In summary, for the first time, the antimicrobial and anticancer effects of a *B. macrophylla* ethanol extract were evaluated. The ethanol extract of *B. macrophylla* revealed a broad-spectrum activity against almost all the tested microorganisms and effectively inhibited cancer cell viability. Further investigation is necessary to discover the mechanisms mediating these antimicrobial and anticancer activities and identify pathways to be targeted for therapeutic applications.

## Figures and Tables

**Table 1:** Qualitative phytochemical screening of the *Bouea macrophylla* extract tested in this work.

Phytochemical Constituents	Test Name	Results
Polyphenol	FeCl_3_ test	**+++**
Tannins	Gelatin test	**++**
Flavonoids	Alkali test	**++**
Carbohydrates	Benedict ’s testFehling’s test	**++** **++**
Steroids	Liebermann–Burchard reaction	**−**
Triterpenoid	Salkowski’s test	**++**
Fixed oils	Spot Tests	**+**
Saponins	Frothing test	**++**
Alkaloids	Mayer’ testWagner’s test	**++** **++**

**+++** Appreciable amount (positive within 2 min..); **++** Moderate amount (positive after 2 min. but within 5 min.); **+** Trace amount (positive after 5 min. but within 10 min.); **−** Completely absent.

**Table 2:** GC–MS-identified compounds from the *B. macrophylla* extract.

Peak No.	Retention Time (min.)	Putative Identity	% Area	MW (Da)	*m/z*	Elemental Composition	Biological Activity
**1**	15.603	Caryophyllene	27.484	204	93, 105, 120, 133, 161, 189	C_15_H_24_	Antifungal [27], anticancer [28], antioxidant, anti-inflammatory, and antimicrobial [28,29]
**2**	16.091	Humulene	3.871	204	93, 107, 121, 147, 175, 189	C_15_H_24_	Antibacterial [30], anti-inflammatory [31]
**3**	17.866	Caryophyllene oxide	5.75	220	79, 93, 121, 149, 177, 205	C_15_H_24_O	Antifungal [27], anticancer [29]
**4**	20.723	2-Methyl-cis-7,8-epoxynonadecane	1.603	296	57, 97, 127, 197, 239, 281	C_20_H_40_O	Unknown
**5**	22.326	Hexadecanoic acid, ethyl ester	2.718	284	88, 101, 157, 213, 239, 300	C_18_H_36_O_2_	Anti-inflammatory, anticancer, and hepatoprotective [32]
**6**	23.54	Phytol	2.791	296	71, 123, 151, 196, 278, 300	C_20_H_40_O	Anticancer, antioxidant, anti-inflammatory, diuretic, antitumor, chemopreventive, antimicrobial, use in vaccine formulations [33,34,35]
**7**	24.08	Oxiraneundecanoic acid, 3-pentyl-, methyl ester, *trans*-	1.132	312	74, 213, 155, 199, 227, 312	C_19_H_36_O_3_	Unknown
**8**	24.309	Hexadecanoic acid, ethyl ester	0.752	284	88, 101, 157, 199, 239, 267	C_15_H_36_O_2_	Antioxidant, nematicidal activities, and hypocholesterolemic [36]
**9**	27.356	Diisooctyl phthalate	5.232	390	104, 149, 210, 279, 330, 360	C_24_H_38_O_4_	Antimicrobial [37,38], inhibiting melanogenesis [39]
**10**	29.789	Squalene	32.114	410	69, 5, 191. 257, 341, 367	C_30_H_50_	Antioxidant, antitumor, and cytoprotective effects [40,41]
**11**	30.808	*trans*-Geranylgeraniol	1.725	290	41, 69, 93, 136, 189, 272	C_20_H_34_O	Anticancer [42,43], antimicrobial [44]
**12**	33.581	Vitamin E	4.889	430	71, 165, 205, 295, 368, 415	C_29_H_50_O_2_	Antioxidant and anti-inflammatory [45], antimicrobial [46]
**13**	37.166	Retinol, acetate	6.351	328	43, 69, 119, 197, 253, 268	C_22_H_32_O_2_	Antioxidant [47]
**14**	37.895	γ-himachalene	3.587	284	41, 93, 119, 133, 161, 189	H_15_H_24_	Antioxidant [48]

**Table 3:** Antimicrobial screening test of the ethanolic leaf extract of *B. macrophylla* using various bacterial strains.

Bacterial Strains		Inhibition Zones (mm)						Ciprofloxacin
		*500 mg/mL*	*100 mg/mL*	*10 mg/mL*	*1 mg/mL*	*100 µg/mL*	*10 µg/mL*	*500 µg/mL*
Gram (−)	*Escherichia coli*	20.50 ± 0.60	17.20 ± 0.31	11.50 ± 0.50	No antimicrobial activity			30.33 ± 1.15
	*Shigella flexneri*	22.50 ± 0.50	17.83 ± 0.29	15.5 ± 0.83	No antimicrobial activity			33.50 ± 0.86
	*Vibrio cholerae*	22.16 ± 1.25	17.50 ± 0.50	13.16 ± 1.26	11.17 ± 0.29	No antimicrobial activity		28.33 ± 0.29
	*Pseudomonas* *aeruginosa*	16.66 ± 0.29	14.83 ± 0.29	12.83 ± 0.76	No antimicrobial activity			25.42 ± 0.41
Gram (+)	*Staphylococcus* *aureus*	12.86 ± 0.15	No antimicrobial activity					18.66 ± 1.54
	*Listeria* *monocytogenes*	17.83 ± 0.76	16.16 ± 1.04	14.83 ± 0.29	13.5 ± 0.50	11.50 ± 0.50	Not observed	30.66 ± 0.58
	*Bacillus cereus*	24.83 ± 0.29	22.5 ± 0.5	17.83 ± 0.29	18.17 ± 0.29	17.5 ± 0.5	17.83 ± 0.76	26.12 ± 0.15
	*Enterococcus faecalis*	16.00 ± 0.50	13.00 ± 0.60	No antimicrobial activity				20.66 ± 0.29
	*Streptococcus mutans*	No antimicrobial activity						23.66 ± 0.29
Mycete	*Candida albicans*	21.5 ± 1.3	21 ± 1	17.83 ± 0.76	11.83 ± 0.76	No antimicrobial activity		25.66 ± 0.34

## Supplementary Materials

The following are available online at https://www.mdpi.com/1420-3049/25/8/1996/s1, Figure S1: Peak assignment identified from Caryophyllene by GC-MS method.; Figure S2: Peak assignment identified from Humulene by GC-MS method.; Figure S3: Peak assignment identified from Caryophyllene oxide by GC-MS method.; Figure S4: Peak assignment identified from 2-Methyl-cis-7,8-epoxynonadecane by GC-MS method.; Figure S5: Peak assignment identified from Hexadecanoic acid, ethyl ester by GC-MS method.; Figure S6: Peak assignment identified from Phytol, ethyl ester by GC-MS method.; Figure S7: Peak assignment identified from Oxiraneundecanoic acid, 3-pentyl-, methyl ester, *trans-,* ethyl ester by GC-MS method.; Figure S8: Peak assignment identified from hexadecanoic acid, ethyl ester by GC-MS method.; Figure S9: Peak assignment identified from Diisooctyl phthalate, ethyl ester by GC-MS method.; Figure S10: Peak assignment identified from Squalene by GC-MS method.; Figure S11: Peak assignment identified from trans-Geranylgeraniol by GC-MS method.; Figure S12: Peak assignment identified from Vitamin E by GC-MS method.; Figure S13: Peak assignment identified from Retinol, acetate by GC-MS method.; Figure S14: Peak assignment identified from γ-himachalene by GC-MS method.
