# Supplementary material for: Potential Antimicrobial and Anticancer Activities of an Ethanol Extract from Bouea macrophylla
Source: Molecules. 2020 Apr 24;25(8):1996. doi: 10.3390/molecules25081996 (PMC7221966; doi:10.3390/molecules25081996)
Supplement: Supplementary file 1 [file molecules-25-01996-s001.pdf]

Potential Antimicrobial and Anticancer Activities of Ethanol Extract from *Bouea macrophylla*

Ngoc Hong Nguyen [1], Thuy Trang Nguyen [2], Phu Cuong Ma [3], Qui Thanh Hoai Ta [4], Thuc Huy Duong [5], Van Giau Vo [6,7,*]

[1] *CirTech Institute, Ho Chi Minh City University of Technology, Ho Chi Minh City 700000 and Vietnam.*
[2] *Faculty of Pharmacy, Ho Chi Minh City University of Technology, Ho Chi Minh City 700000, Vietnam.*
[3] *Institute of Applied Science, HCMC University of Technology (HUTECH), Ho Chi Minh City 700000, Vietnam.*
[4] *Institute of Research and Development, Duy Tan University, Danang 550000, Vietnam.*
[5] *Department of Organic Chemistry , University of Education, Ho Chi Minh City 700000, Vietnam.*
[6] *Bionanotechnology Research Group, Ton Duc Thang University, Ho Chi Minh City 700000, Vietnam.*
[7] *Faculty of Pharmacy, Ton Duc Thang University, Ho Chi Minh City 700000, Vietnam*
* *Correspondence: vovangiau@tdtu.edu.vn*

Supplementary material

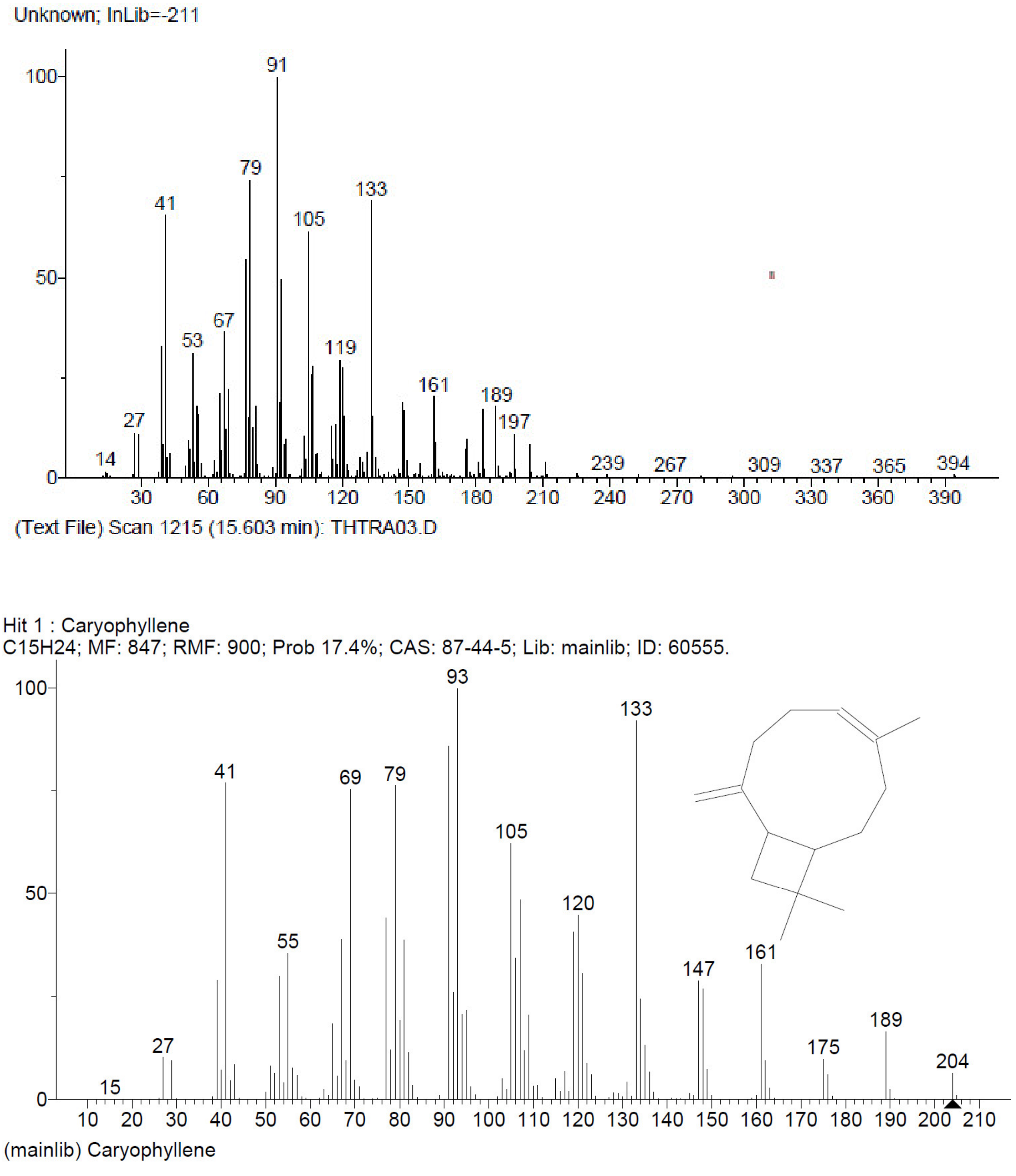

Figure S1. Peak assignment identified from Caryophyllene by GC-MS method.

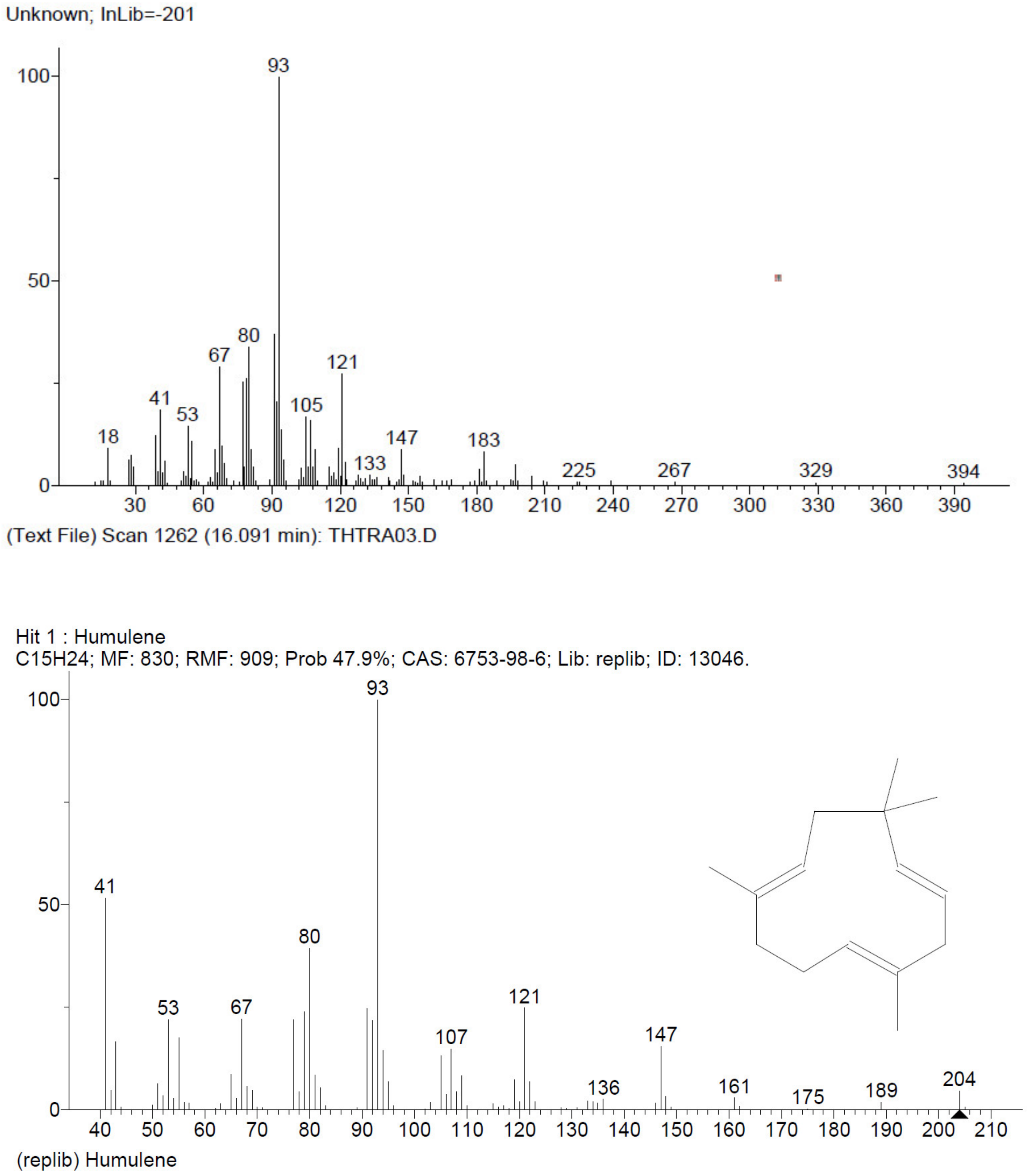

Figure S2. Peak assignment identified from Humulene by GC-MS method.

.

Unknown; InLib=-151

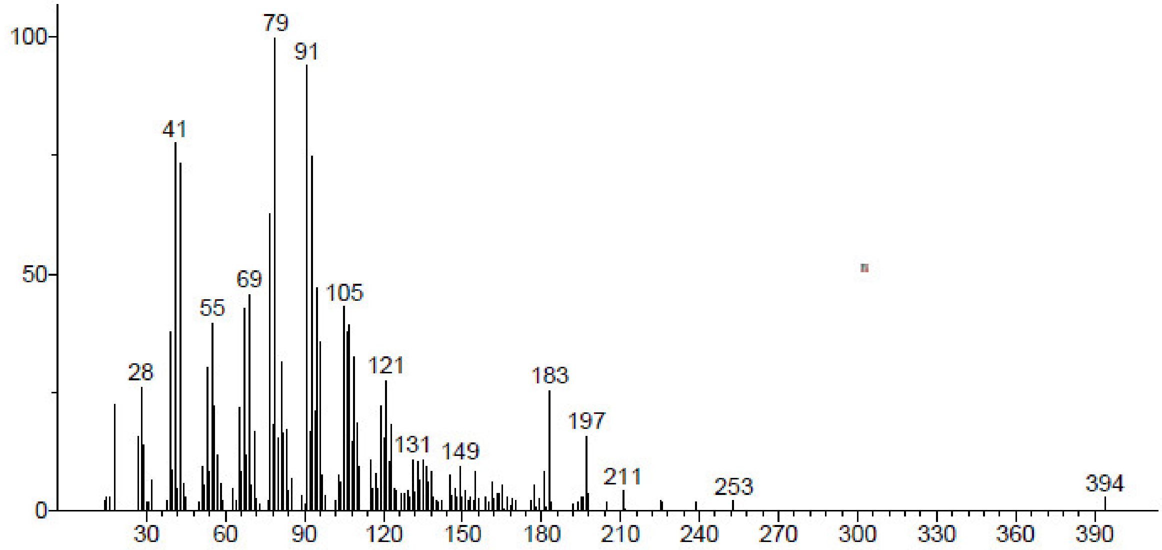

Hit 1 : Caryophyllene oxide
C15H24O; MF: 820; RMF: 884; Prob 35.3%; CAS: 1139-30-6; Lib: mainlib; ID: 5928.

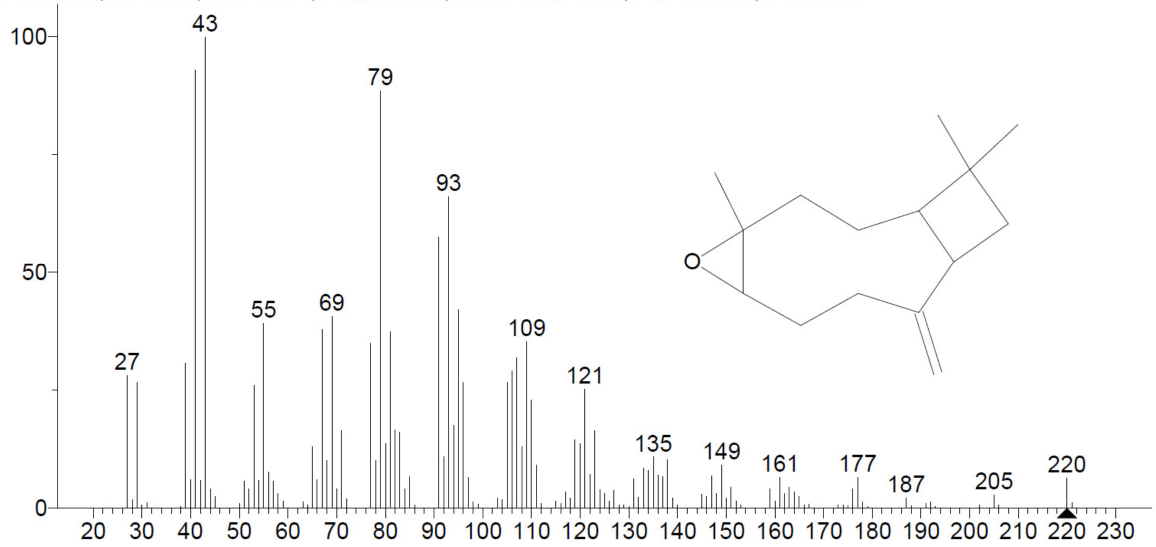

(mainlib) Caryophyllene oxide

Figure S3. Peak assignment identified from Caryophyllene oxide by GC-MS method

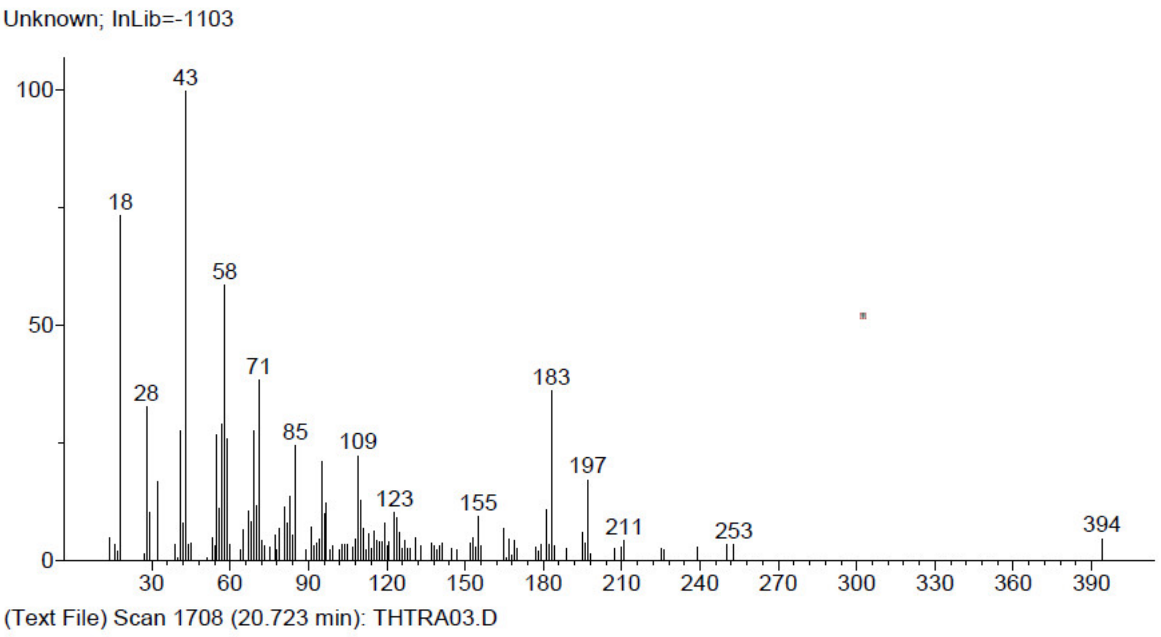

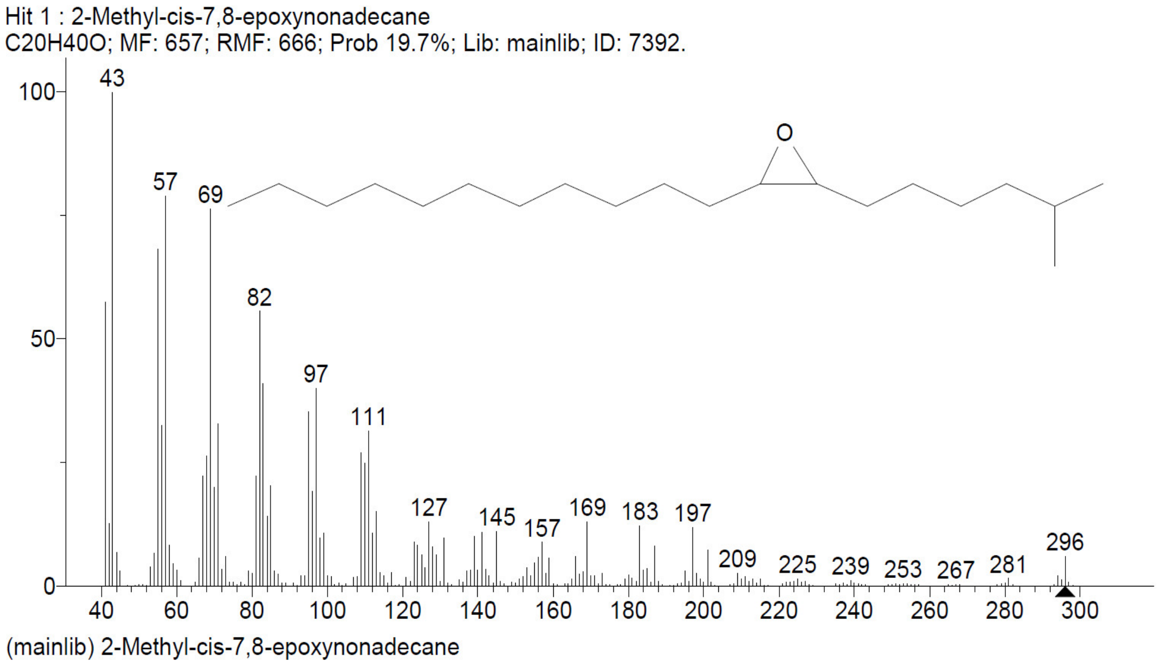

Figure S4. Peak assignment identified from 2-Methyl-cis-7,8-epoxynonadecane by GC-MS method.

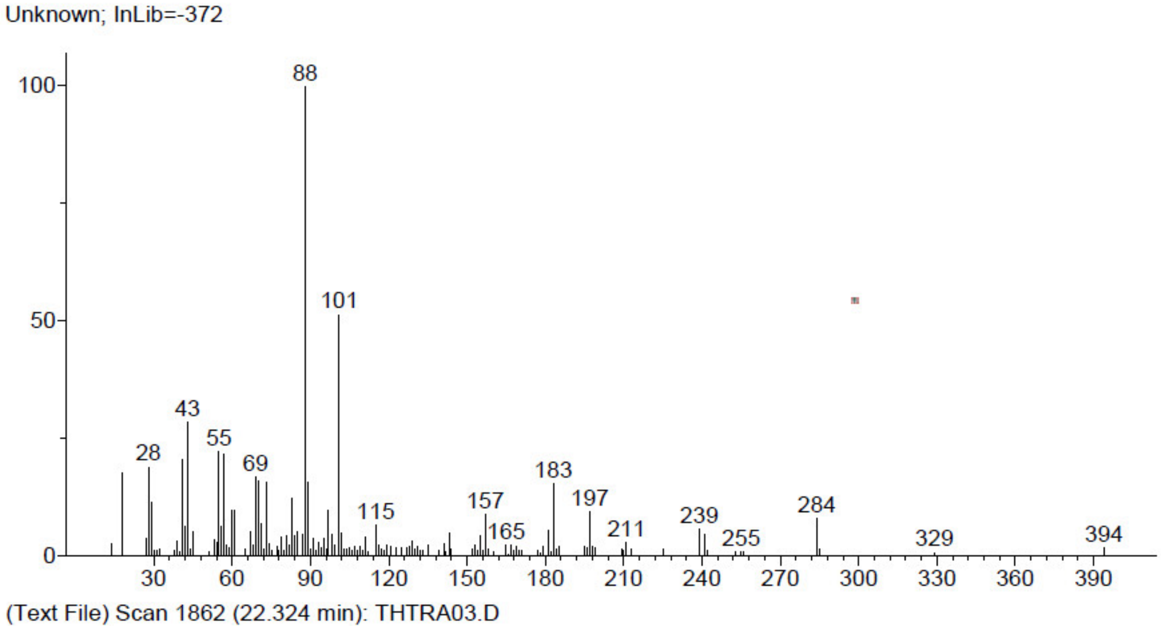

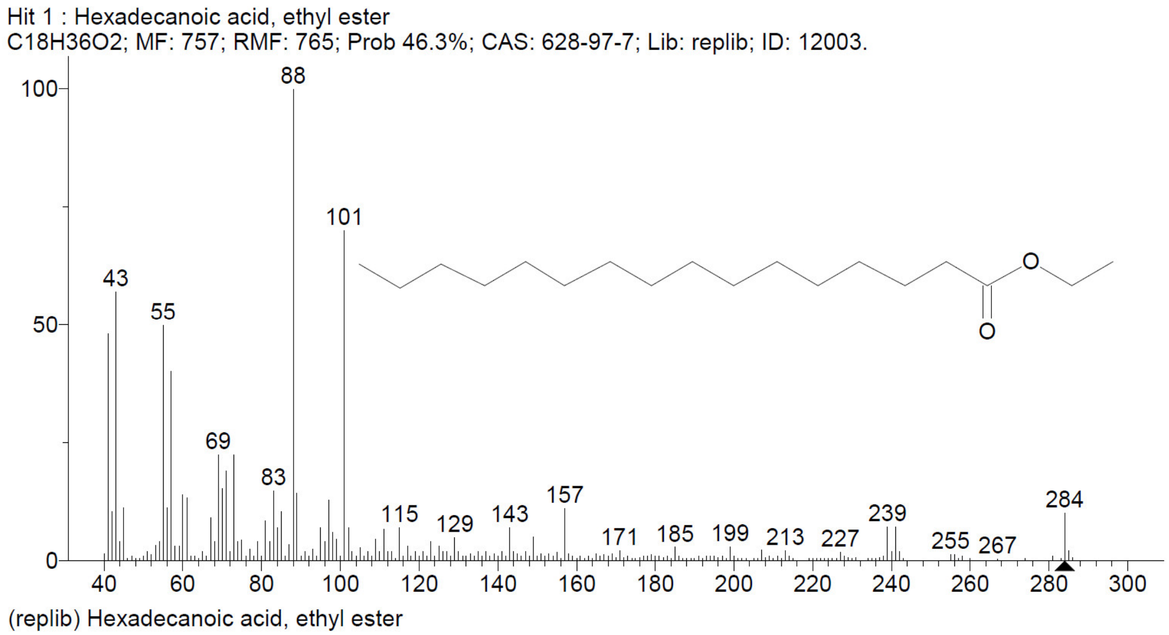

Figure S5. Peak assignment identified from Hexadecanoic acid, ethyl ester by GC-MS method.

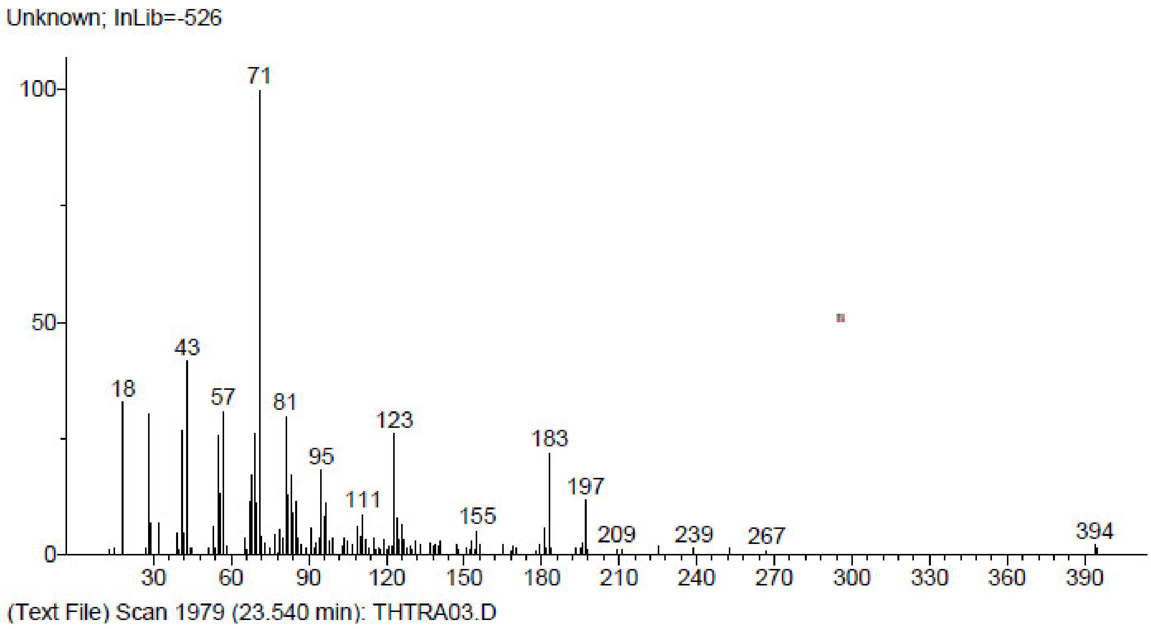

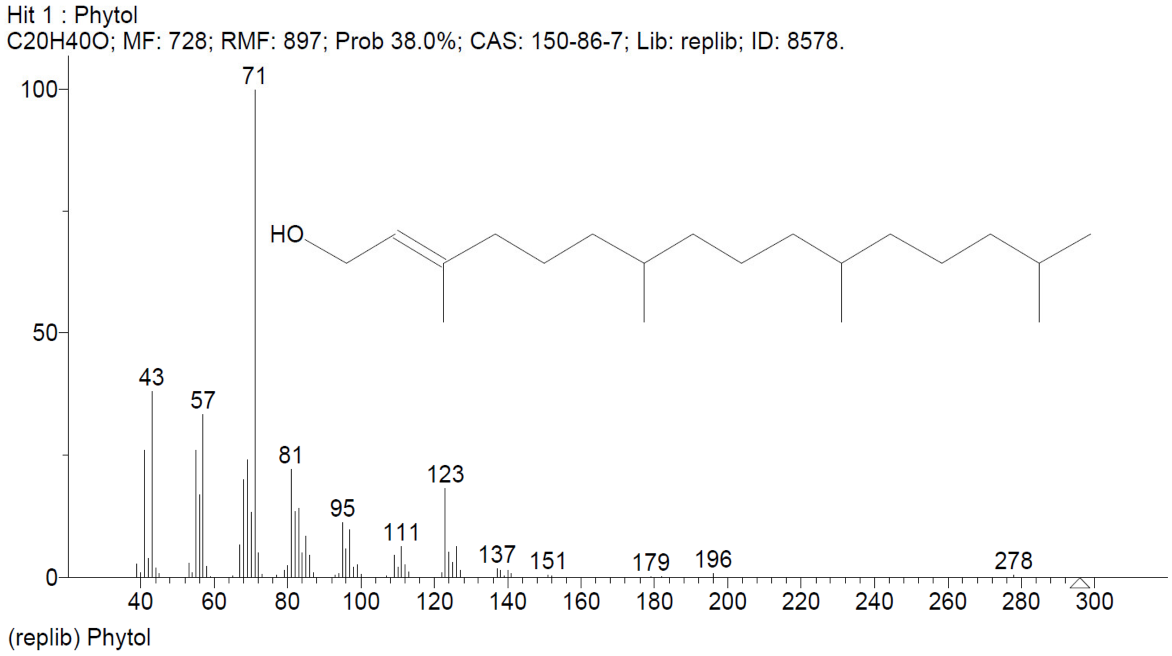

Figure S6. Peak assignment identified from Phytol, ethyl ester by GC-MS method

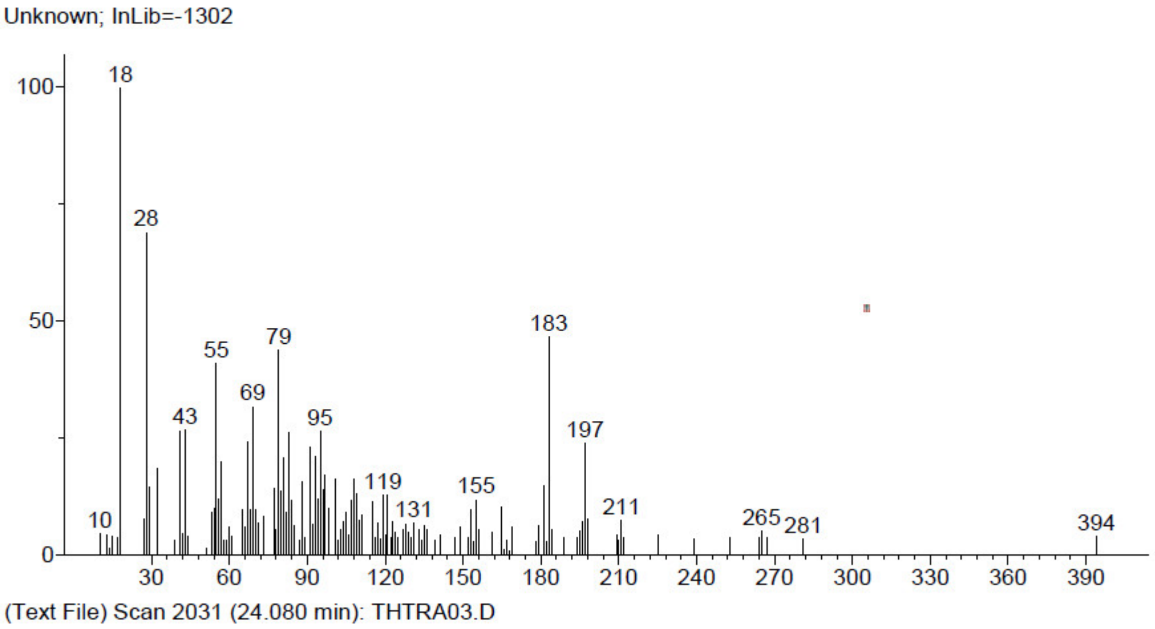

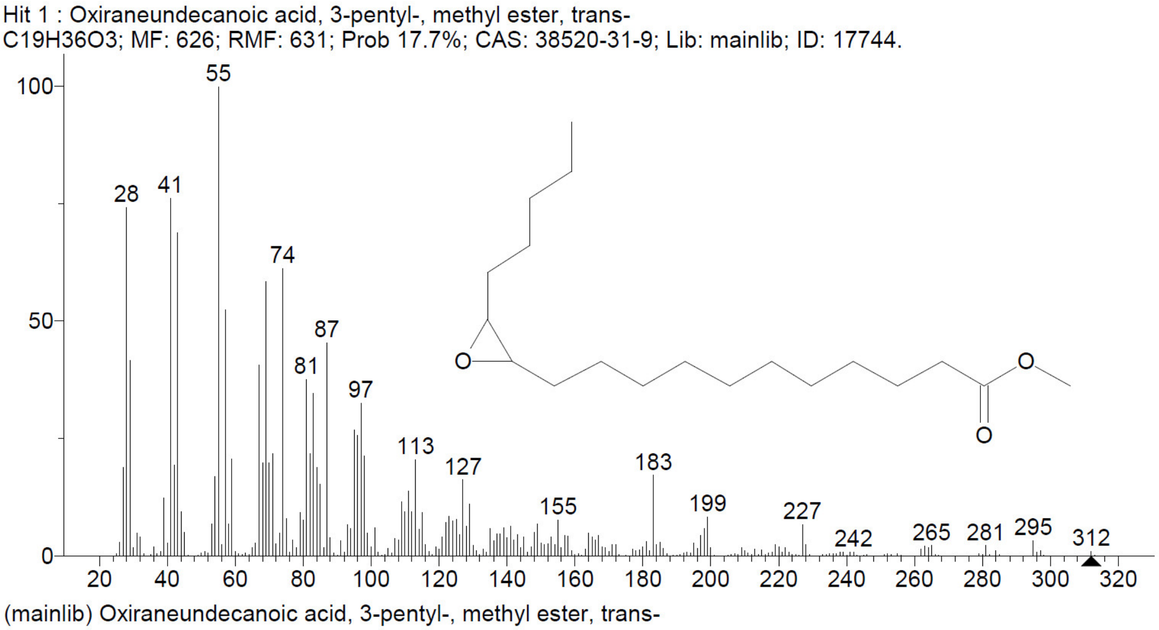

Figure S7. Peak assignment identified from Oxiraneundecanoic acid, 3-pentyl-, methyl ester, *trans*-, ethyl ester by GC-MS method

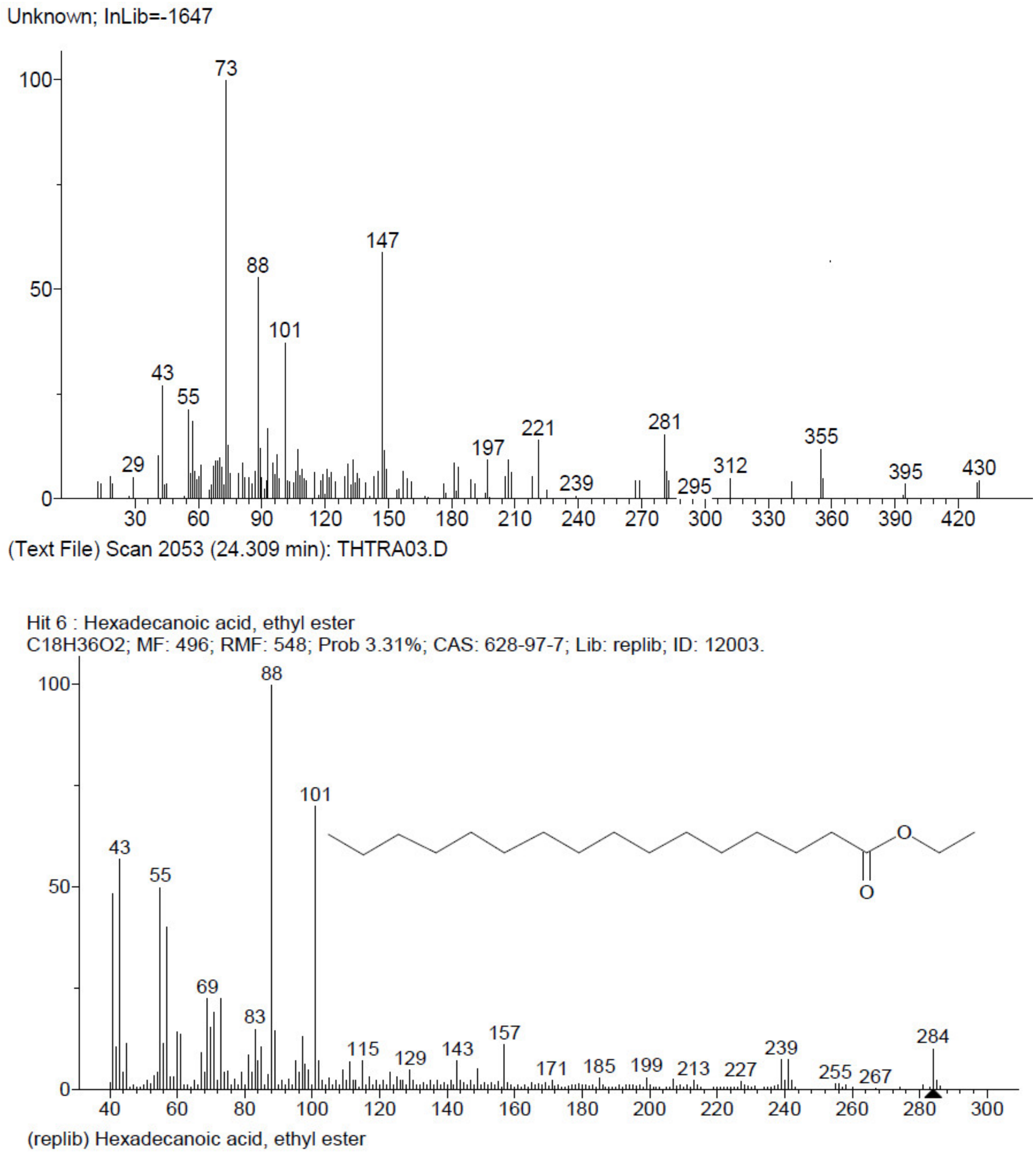

Figure S8. Peak assignment identified from hexadecanoic acid, ethyl ester by GC-MS method

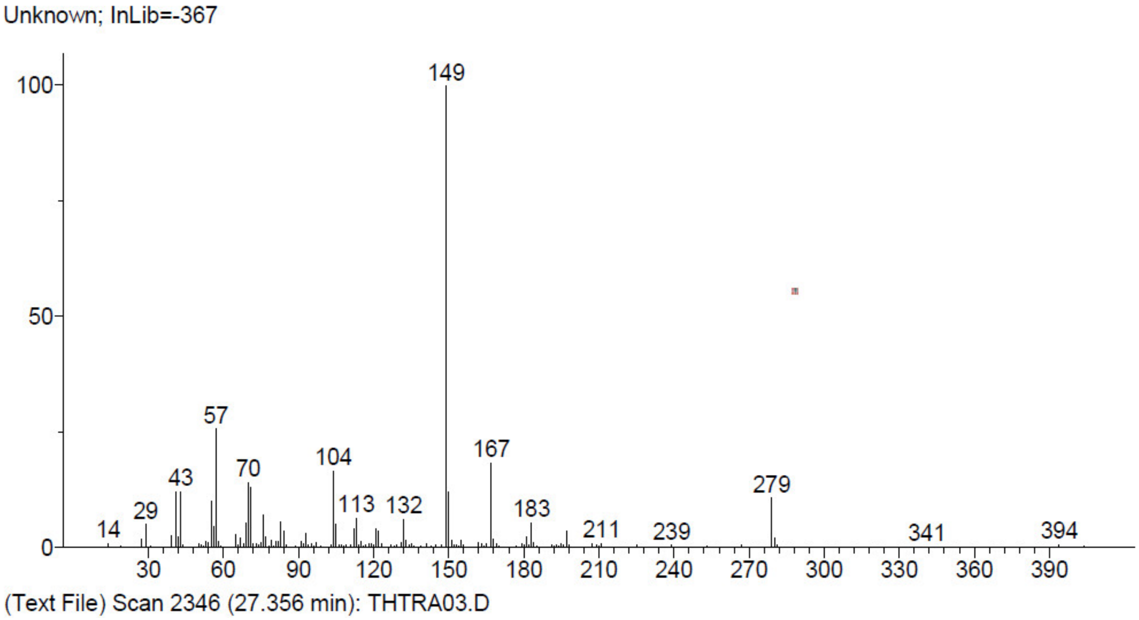

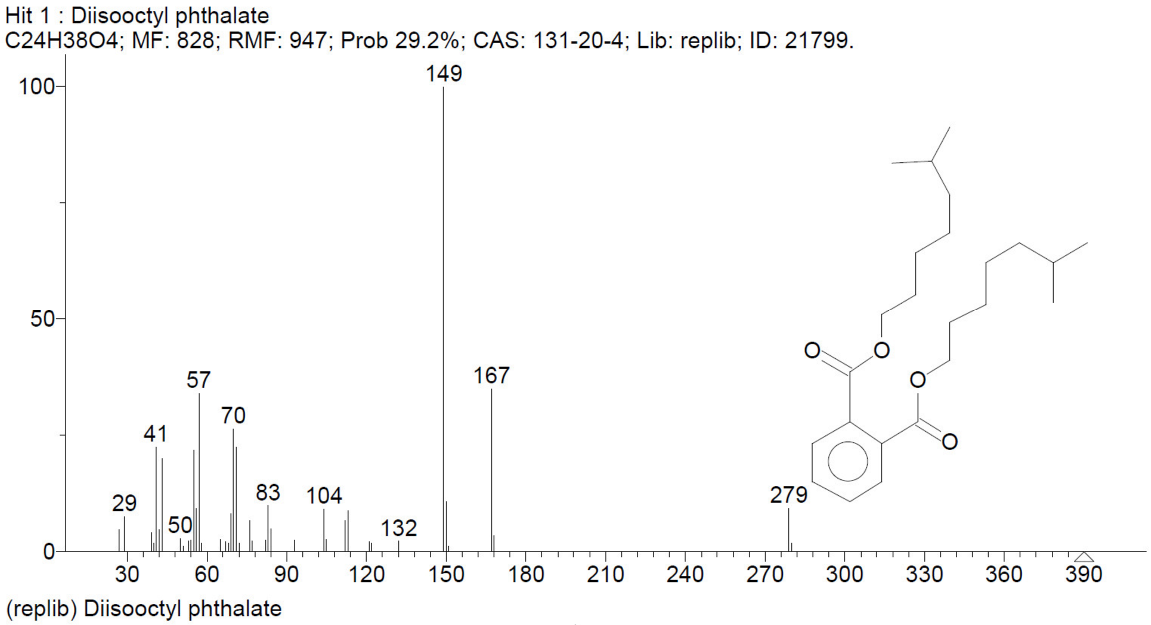

Figure S9. Peak assignment identified from Diisooctyl phthalate, ethyl ester by GC-MS method

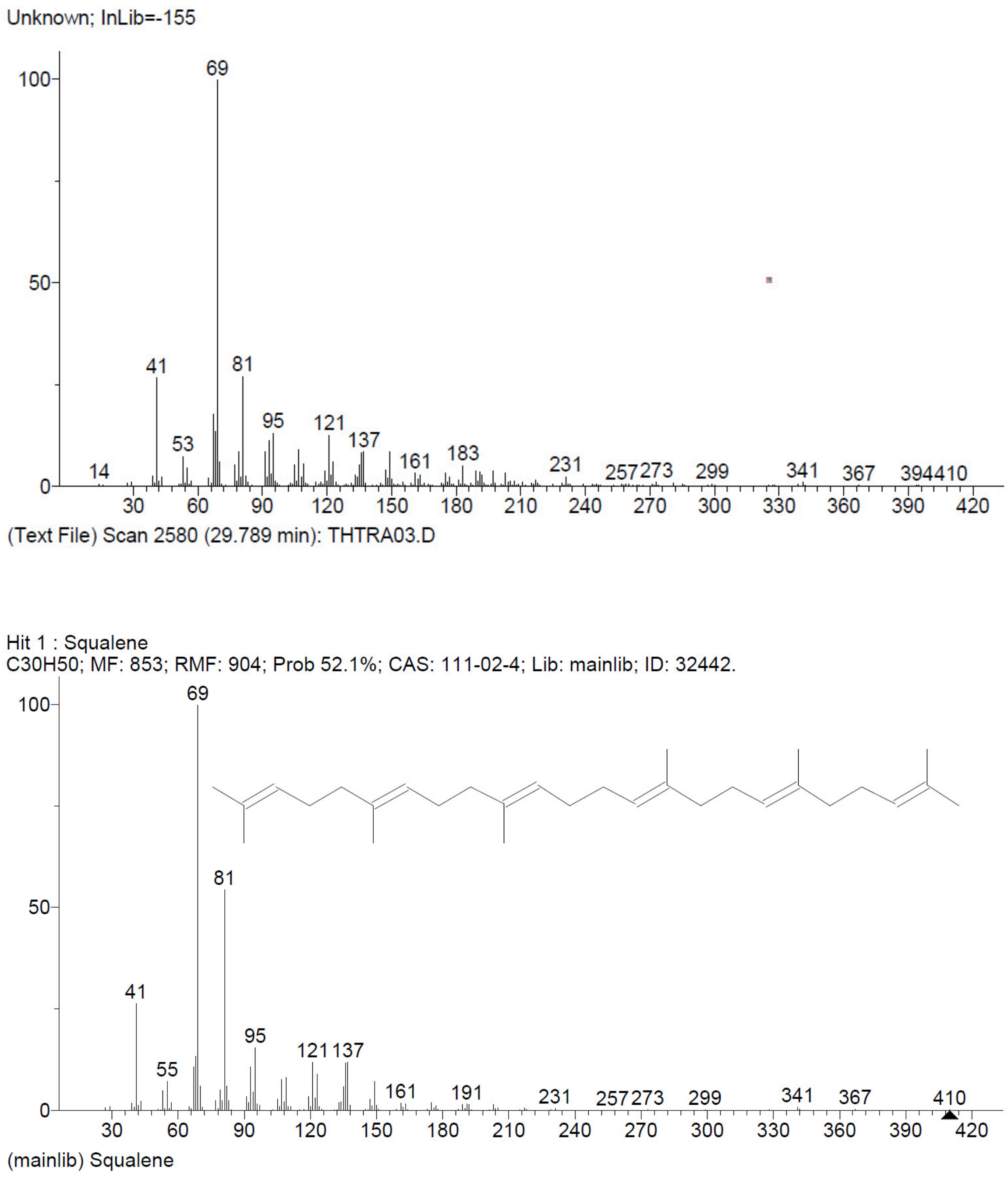

Figure S10. Peak assignment identified from Squalene by GC-MS method

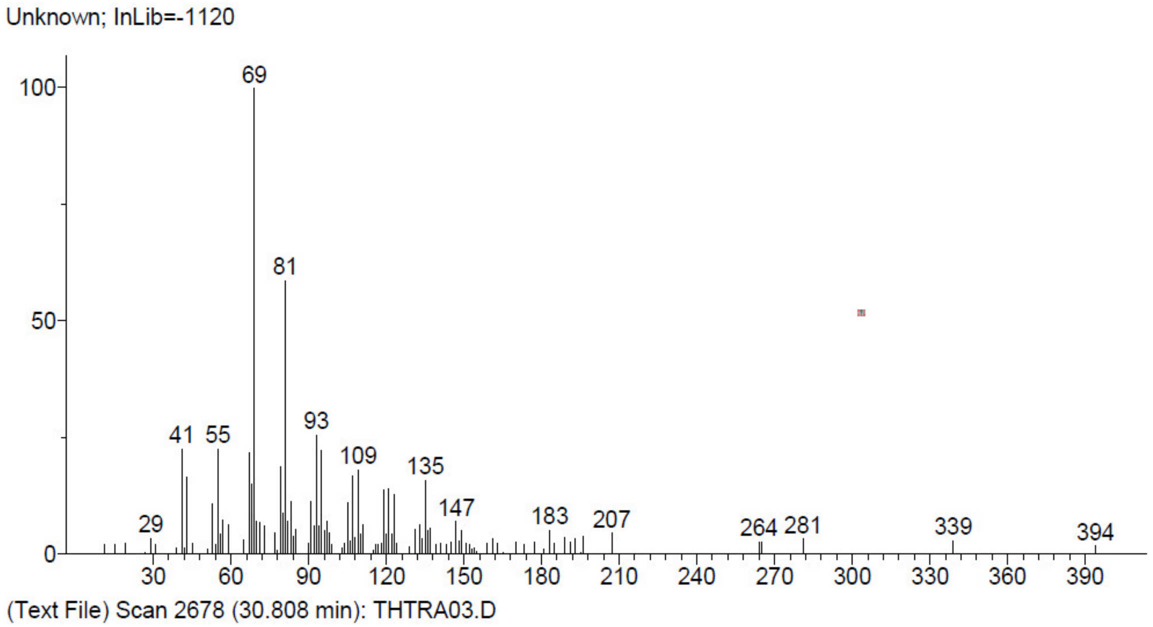

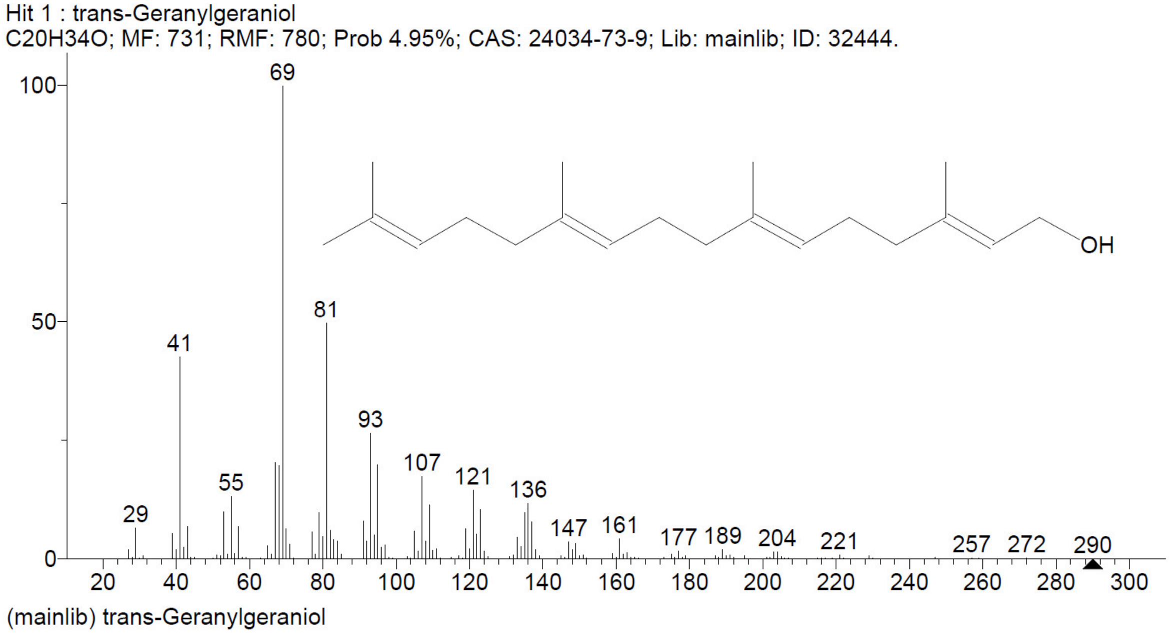

Figure S11. Peak assignment identified from *trans*-Geranylgeraniol by GC-MS method

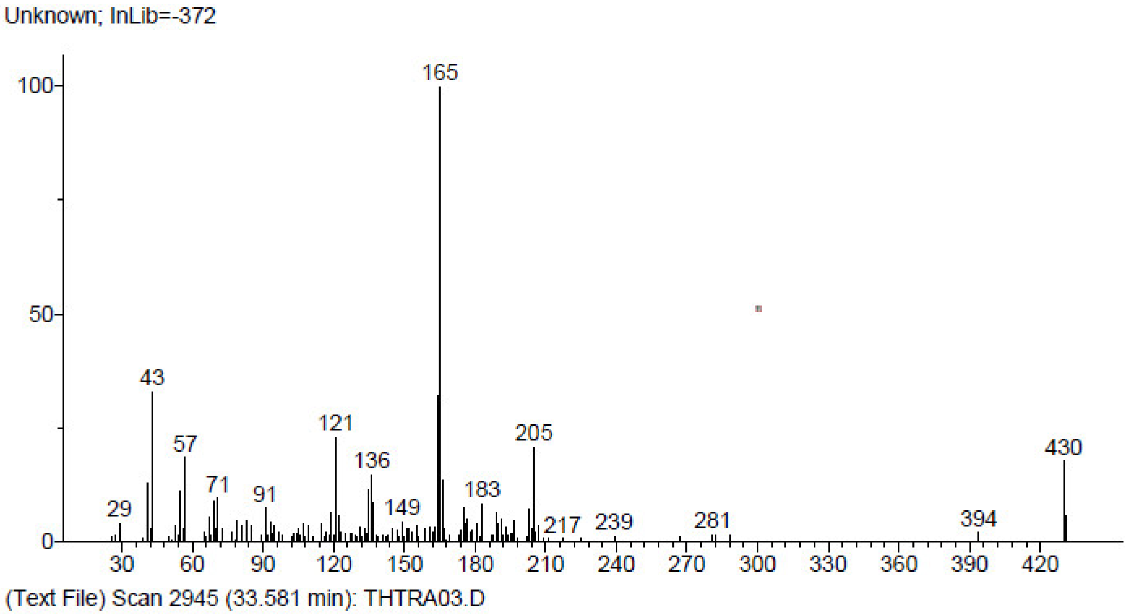

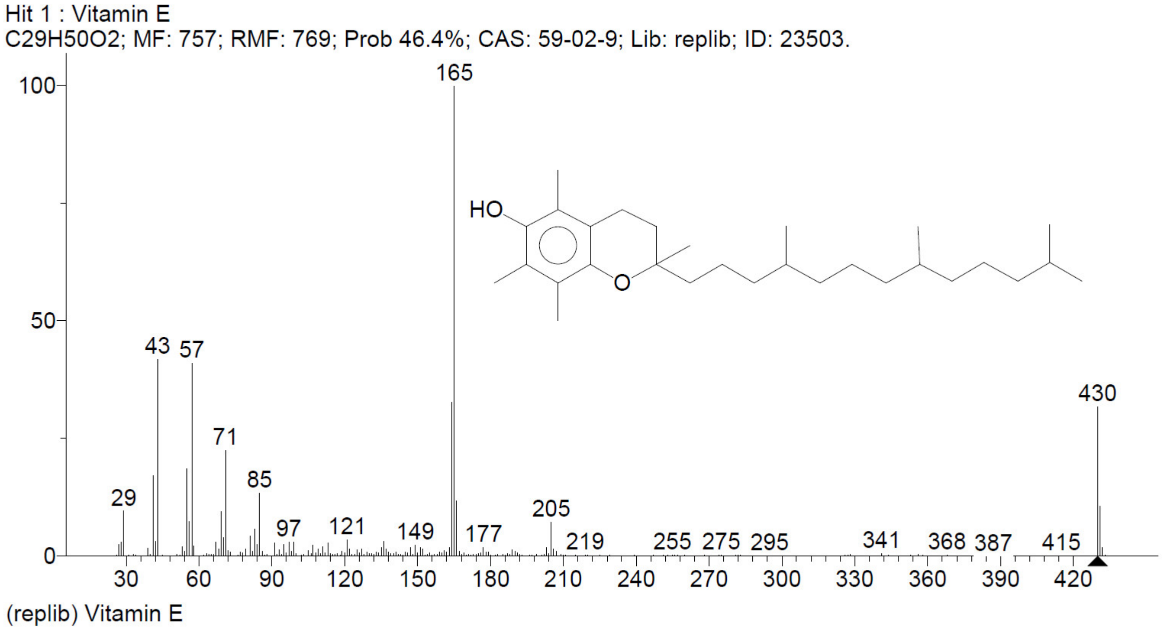

Figure S12. Peak assignment identified from Vitamin E by GC-MS method

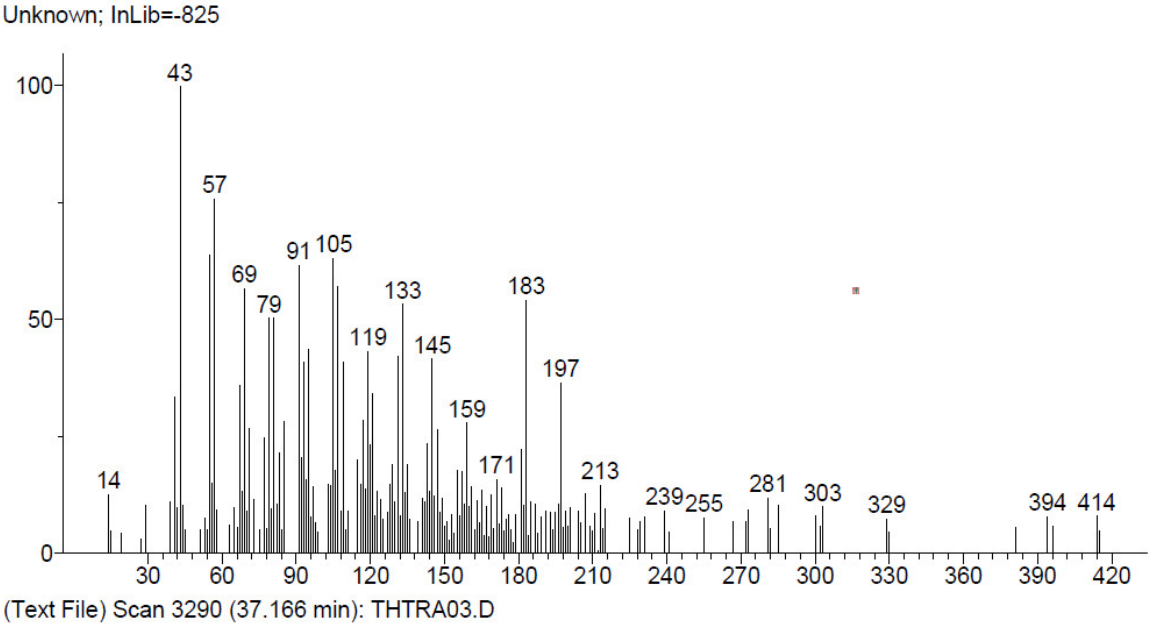

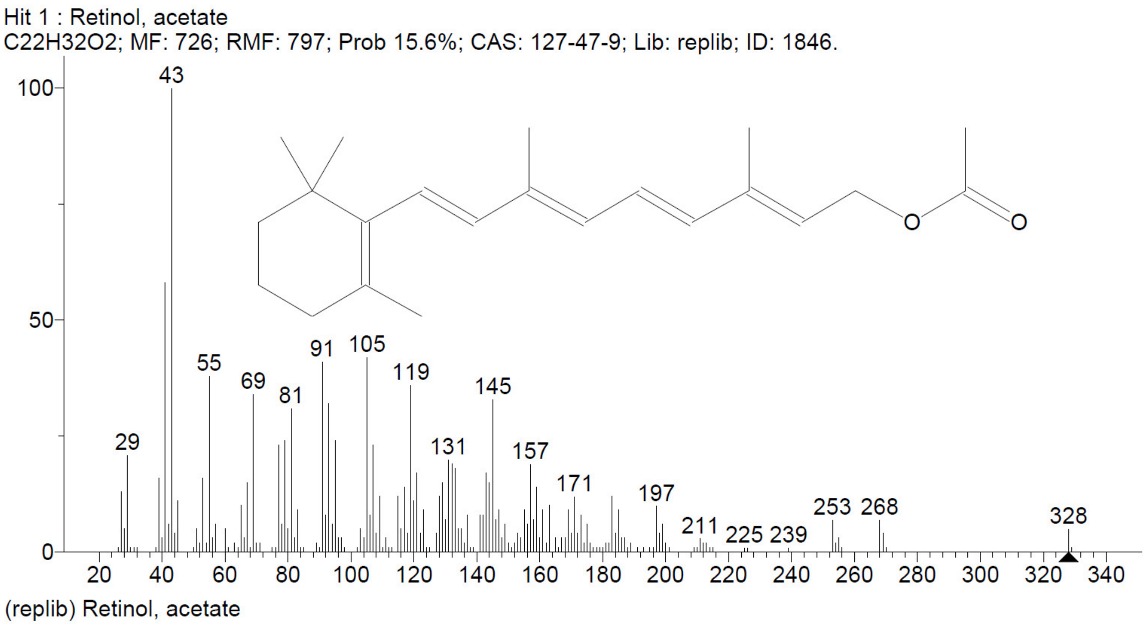

Figure S13. Peak assignment identified from Retinol, acetate by GC-MS method

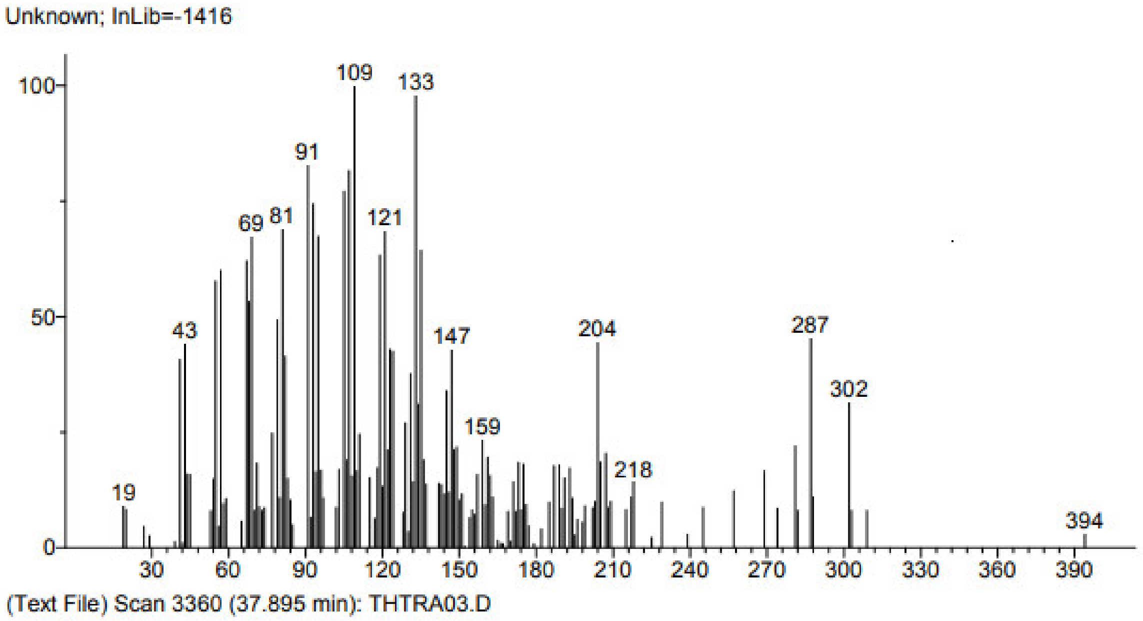

140 m/z Values and Intensities:

19	91	20	82	27	46	29	25	39	13
41	406	42	10	43	444	44	159	45	158
53	79	54	148	55	576	56	46	57	600
58	95	59	106	65	57	67	620	68	532
69	672	70	80	71	182	72	88	73	80
74	86	77	247	79	492	80	108	81	689
82	414	83	149	84	103	85	50	91	830
92	65	93	743	94	164	95	673	96	167
97	107	102	87	103	169	105	770	106	190
107	815	108	155	109	999	110	166	111	245
115	151	116	1	117	63	118	173	119	632
120	132	121	688	122	211	123	429	124	424
128	76	129	269	130	35	131	376	132	142
133	980	134	310	135	642	136	190	137	137
142	139	143	136	144	116	145	339	146	120
147	430	148	212	149	217	150	103	151	116
152	3	154	64	155	81	156	72	157	158
159	235	160	92	161	195	162	156	163	109
165	16	166	9	167	8	169	78	170	14
171	142	172	78	173	184	174	81	175	180
176	93	177	47	179	8	182	40	185	98
187	177	189	179	190	85	191	150	193	172
194	107	195	27	196	60	198	55	199	90
202	86	203	100	204	446	205	185	207	204
208	87	209	100	215	82	217	109	218	143
225	22	229	98	239	28	245	87	257	123
269	166	274	85	281	219	282	80	287	454
288	110	302	314	303	80	309	80	394	32

Figure S14. Peak assignment identified from γ-himachalene by GC-MS method **(*continuing*)**

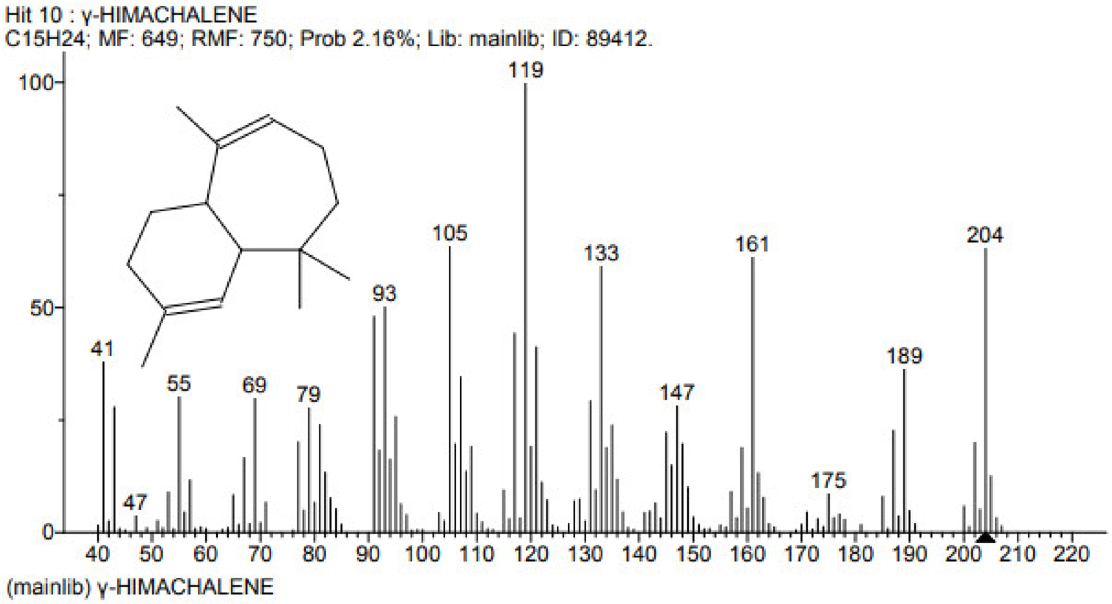

Figure S14. Peak assignment identified from γ-himachalene by GC-MS method
